# Bone Development and Regeneration 2.0

**DOI:** 10.3390/ijms24108761

**Published:** 2023-05-15

**Authors:** Kazuo Yudoh, Yodo Sugishita, Yuki Suzuki-Takahashi

**Affiliations:** Department of Frontier Medicine, Institute of Medical Science, St. Marianna University School of Medicine, Kawasaki 216-8511, Japan; yodo@marianna-u.ac.jp (Y.S.); y2takahashi@marianna-u.ac.jp (Y.S.-T.)

Bone is an important tissue which is a structural body component, carrying out the roles of mechanical stress response and organ/tissue protection. In addition, bone, as an integral organ, not only regulates bone metabolism and regulates the hematopoietic niche, but also acts as an endocrine organ to control some metabolic processes that are independent of bone metabolism.

Bone homeostasis is regulated and maintained by the remodeling cycle of osteoblastic bone formation and osteoclastic bone resorption [1,2]. When breaking the balance of bone homeostasis, bone remodeling cannot maintain an invariant bone mass, consequently leading to osteopenia and eventually osteoporosis [1,2,3,4]. Osteoporosis is an age-related common disease that is characterized by low bone mass and bone microstructural destruction resulting from the downregulation of bone remodeling and bone homeostasis [1,3,4]. In comparison with other organs/tissues, bone shows a high regenerative and remodeling potential throughout the human lifespan. Although bone tissue has a high regenerative and remodeling potential, this gradually decreases with age. Indeed, with the continual extension of life expectancy, aging-related bone mass loss and pathologies are increasing in a gradual manner, which negatively influences the quality of daily living of an increasing number of individuals. In particular, osteoporosis causes significant impacts on activities of daily living (ADL), which are expected to be further accentuated in future by the continuous increase in life expectancy. New scientific capabilities and perspectives are needed to further understand the mechanism of bone homeostasis.

Regarding bone metabolism, several mechanisms and pathways, such as the wingless-type (WNT)/beta-catenin, bone morphogenetic protein (BMP) 2 or parathyroid hormone (PTH) signaling pathways, have been thoroughly studied over the last few decades. Among numerous regulatory factors in bone metabolism, mechanical stress is recognized as a critically important factor in bone-associated cell differentiation/growth and their functions, including those of osteoblasts, osteoclasts and osteocytes [5,6,7]. Recent studies have demonstrated that physiologic mechanical loading stimulates osteoblast differentiation and resultant bone formation, and this is regulated by the molecular signaling pathway controlling osteocytes in response to mechanical stress [8,9,10]. Lin C. et al. demonstrated that osteocyte-produced sclerostin (in response to mechanical loading) controls the bone-remodeling cycle (bone formation and resorption) as a master molecule in mechano-transduction [11]. Mechanical-stress-mediated bone metabolism is realized through the interaction between two opponent mechanisms: (1) mechanical unloading accelerates sclerostin expression, which counteracts the Wnt/beta-catenin signaling pathway through the interaction between osteocytes and osteoblasts, allowing the concurrent Wnt-noncanonical pathway in osteocytes to osteoclasts, and is directed at bone resorption; on the contrary, (2) mechanical loading decreases the expression of sclerostin, inducing the activation of Wnt/beta-catenin signaling in osteocytes, consequently resulting in osteoblast differentiation and bone formation [11]. The interaction of osteoblasts with osteocytes through the osteocytic sclerostin-Wnt/beta-catenin signaling pathway is closely implicated in the mechanical-stress-mediated osteoblast differentiation following the acceleration of bone formation [8,9,10,11,12].

Conversely, a recent report clearly indicates that physiologic mechanical stress directly causes osteoblast differentiation and increased bone formation without a mechanism involving the Wnt/beta-catenin signaling pathway connecting osteocytes and osteoblasts as mentioned above [13]. Somemura S. et al. studied the interaction of the mechanical stress response with glucose metabolism via the glucose transporter (Glut)-1 and energy sensor sirtuin (Sirt)-1 in osteoblast energy metabolism [13]. They clearly revealed that both regulators of energy metabolism, Glut-1 and Sirt-1, also function as master molecules of stress responses against mechanical loading in osteoblasts. Mechanical loading to osteoblasts changed the expression of Glut-1 and Sirt-1 following the activation of the osteogenic transcription factor, Runx2, and resultant bone formation. Indeed, the inactivation of cell surface Glut-1 by the inhibitor significantly reduced the mechanical-loading-induced changes in the Sirt-1-to-Runx2 signaling pathway as well as bone formation activity, suggesting that the activation of Glut1 is required for mechanical-stress-mediated osteoblast differentiation and bone formation, via the signal transduction network between the energy sensor Sirt-1 and the osteogenic transcription factor Runx2, in osteoblasts. The Glu-1-Sirt-1-Runx2 pathway in osteoblasts may play some sort of role in mechanical-stress-mediated bone formation and osteoblast differentiation, without the osteocytic sclerostin−Wnt/beta-catenin signaling pathway. Mechanical loading may directly induce osteoblast differentiation and bone formation through the signaling pathway of glucose metabolism, without a sclerostin–Wnt/beta-catenin-dependent pathway through osteocyte-to-osteoblast contact. Indeed, it has been indicated that glucose uptake induces the osteoblast differentiation and bone formation potential by activating Runx2 activity in osteoblasts [14,15,16,17].

Attention has been attracted by recent findings that the nicotinamide adenine dinucleotide (NAD)-dependent deacetylase Sirt-1 regulates many metabolic functions, such as inflammatory response, apoptosis, cell cycle, DNA repair, genome stability, mitochondrial function, cellular energy metabolism (adenosine triphosphate production) and cell responses to extrinsic stresses, including mechanical stress [18,19,20,21,22,23]. It has been also indicated that Sirt-1 has two important roles—“regulation of cell energy metabolism” and “response to cellular stresses (stress tolerance)”—which are involved in some metabolisms, and the pathogenesis and pathology of a variety of diseases, including mechanical-stress-induced degenerative diseases [24,25,26]. These findings provide evidence to support Sirt-1 activity being a key factor which links the mechanical stress response to energy metabolism in osteoblasts. In other words, the cell surface Glut-1-to-Sirt-1 pathway may have an important role as a mechano-sensor in osteoblasts. Further understanding of the mechanisms involved in the response of osteoblasts to mechanical stress is conducive to the elucidation of bone metabolism and the pathophysiology of osteoporosis.

In particular, several factors and proteins are linked to the regulatory mechanisms of bone development and growth, as well as bone regeneration and degeneration [27,28,29,30]. Saito M et al. indicated that 4.1 G, a plasma-membrane-associated cytoskeletal protein, promotes primary ciliogenesis in the differentiating preosteoblasts and induction of cilia-mediated osteoblast differentiation at the newborn stage [27]. Interestingly, it has been demonstrated that LIM-homeodomain transcription factor (Lmx1b), which plays a key role in body pattern formation during development, negatively regulates osteoblast differentiation and function through regulation of Runx2 [28]. Although further studies are needed to clarify the exact mechanism in bone biology, these papers will generate a representative picture of the latest advances in bone research and serve as a road map for where the field is headed.
